# A Rare Case of Malignant Syndrome in Parkinson’s Disease Caused by Severe Dehydration and Complicated by Syndrome of Inappropriate Antidiuretic Hormone Secretion (SIADH)

**DOI:** 10.7759/cureus.42377

**Published:** 2023-07-24

**Authors:** Ahmed Ali Aziz, Muhammad Ali Aziz, Rehan Shah

**Affiliations:** 1 Internal Medicine, Saint Francis Medical Center, Trenton, USA; 2 Internal Medicine, Bronxcare Health System, New York, USA

**Keywords:** levodopa-carbidopa, parkinson' s disease, siadh, neuroleptic malignant syndrome, malignant syndrome

## Abstract

Malignant syndrome (MS) in Parkinson’s disease (PD) is a rare complication that occurs in patients who have a history of PD and are taking dopaminergic drugs. The syndrome is quite similar to neuroleptic malignant syndrome (NMS) in presentation and is a potentially fatal syndrome. Awareness of symptoms, early diagnosis, and the ability to differentiate it from NMS is important to prevent mortality. Clinical manifestations of MS are similar to NMS and include altered mentation, rigidity, fever, leukocytosis, and elevated serum creatine kinase (CK). However, MS is differentiated from NMS by the precipitating factors; of which, the commonest precipitating factor for MS is dopaminergic drug withdrawal or dose reduction while other less common causes include infection, dehydration, and hot weather. We present a rare case of MS in a patient with a history of PD precipitated by severe dehydration and hot weather in the absence of dopaminergic drug withdrawal. He presented with fever, severe rigidity, altered mentation, dehydration, leukocytosis, and elevated CK. He was correctly diagnosed with MS and promptly treated, preventing mortality. The triad of fever, severe rigidity, and altered sensorium in a patient with a history of PD should prompt evaluation for MS in addition to NMS to initiate appropriate treatment and prevent mortality.

## Introduction

Malignant syndrome (MS) in Parkinson’s disease (PD) was first described by Toru et al [1]. It is a syndrome occurring during the course of treatment of PD with dopaminergic drugs [1]. It is usually precipitated by the acute withdrawal or dose reduction of dopaminergic/anti-parkinsonian drugs used to treat PD. However, other conditions such as infections, dehydration, and hot weather can also precipitate MS in patients who otherwise had no changes to their dopaminergic dosage for PD [2-5]. Clinical manifestations of MS are similar to those of neuroleptic malignant syndrome (NMS) and include fever, altered levels of consciousness, rigidity, and dysautonomia. Laboratory evaluation shows leukocytosis and elevated serum creatine kinase (CK). It is a potentially fatal syndrome and awareness of its symptoms and early detection are important to prevent mortality. We present a rare case of MS in a PD patient precipitated by severe dehydration and hot weather who was otherwise taking his anti-parkinsonian medications as usual.

## Case presentation

A 65-year-old male with a history of PD was brought to the emergency department for altered mentation, agitation and severe rigidity. He could not provide much history as he was confused. However, his family members told us that he had a history of PD and he was taking levodopa-carbidopa 100 mg-25 mg four times daily for the past four years. He had good compliance with his medications and his parkinsonian symptoms were well controlled on this medication regimen. They also told us that for the past few days, he had been working out in hot weather and had limited water intake. On presentation his heart rate was 142 beats per minute, his blood pressure was 95/58 mm Hg, he was febrile at 101.7 degrees Fahrenheit and he was tachypneic to 22 breaths per minute. His oxygen saturation was 100% on room air. On physical exam, he was confused, had axial and appendicular rigidity, and appeared dehydrated.

His admission labs were significant for serum sodium of 140 mmol/L, creatinine 1.9 mg/dL, CK 1628 U/L, and lactic acid of 7.0 mmol/L. He had leukocytosis of 10.8 x 10^3^ K/uL (Table 1). His urinalysis results were normal except for trace ketones. His urine drug screen was negative for any drug use. His serum ammonia and thyroid-stimulating hormone (TSH) levels were normal. 

**Table 1:** Lab values on admission. BUN: blood urea nitrogen

Lab Parameters (units)	Reference Range	Result
Sodium (mmol/L)	135 – 145	141
Potassium (mmol/L)	3.5 – 5.5	4.3
Chloride (mmol/L)	96 – 106	104
BUN (mg/dL)	6 – 24	20
Creatinine (mg/dL)	0.7 – 1.3	1.98
Bicarbonate (mmol/L)	22 – 29	17
Glucose (mg/dL)	70 - 100	98
Creatine Kinase (U/L)	30 – 223	1628
Ammonia (umol/L)	16 – 53	67
Lactate (mmol/L)	0.5 – 2.0	7.0
White Blood Cells (k/uL)	3.8 – 10.2	10.8
Hemoglobin (g/dL)	12.9 – 16.7	14.6
Hematocrit (%)	39.2 – 48.8	43.3
pH	7.35 – 7.45	7.38
PaO2 (mm Hg)	75 – 100	94
PaCO2 (mm Hg)	35 - 45	38

**Figure 1:**
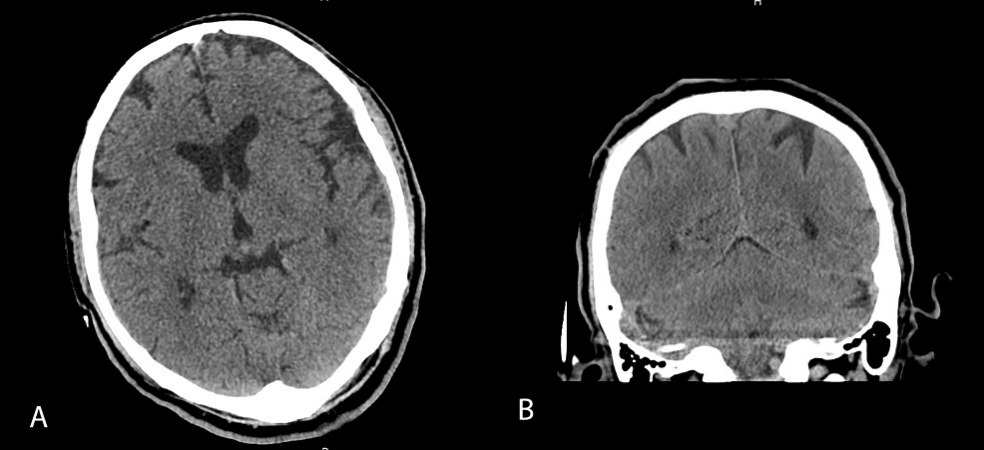
CT scan head without intravenous contrast. A = Transverse view. B = Coronal view

A computed tomography (CT) scan of the brain without intravenous contrast was normal without any acute intracranial abnormalities (Figure 1). A CT scan of the chest abdomen and pelvis was normal without any acute chest, intra-abdominal or pelvic pathology. A lumbar puncture revealed clear-colored cerebrospinal fluid (CSF) and fluid analysis was negative for any bacterial, fungal or viral infection (Table 2).

**Table 2:** Cerebrospinal fluid analysis.

Cerebrospinal Fluid Component (units)	Reference Range	Result
Glucose (mg/dL)	40 – 70	64
Protein (mg/dL)	15 – 45	44
White Blood Cells (/uL)	0 – 5	5
Red Blood Cells (/uL)	2 – 5	3508

The patient was started on intravenous (IV) fluids (0.9% normal saline) and blood cultures were sent for testing. Because infection could not be ruled out, the patient was started prophylactically on antibiotics. His serum lactate levels normalized four hours later after IV fluid hydration to 0.9 mmol/L. As he was compliant with his medications and did not have any recent medication changes nor a history of antipsychotic drug use it was unlikely his symptoms were from NMS although his clinical manifestation was very similar to NMS. Because he was severely dehydrated and was working in hot weather without any change in his anti-parkinsonian medications, a diagnosis of MS in PD was made. 

On Day 2 of hospitalization, his mentation improved. He was more awake, not agitated any more, and was able to converse. He still had axial and appendicular rigidity. He told us that he could not recall exactly what happened to him but the last thing he remembered was that he was working outside and it was hot and he couldn’t find water to drink. He also confirmed that he did not run out of his anti-parkinsonian medications or try any new medication and was taking them as prescribed. On Day 2 his leukocytosis resolved, he did not have any fever spikes, and his CK down trended to 1506 U/L. His blood cultures remained negative. He was re-started on his home anti-parkinsonian medications. Because no infectious source could be identified, blood cultures remained negative, he was afebrile and leukocytosis resolved his antibiotics were discontinued.

On Day 3 labs showed improvement in CK levels to 964 U/L. He was tolerating his anti-parkinsonian medications well. His rigidity markedly improved and he was able to sit in a chair and walk. He was tolerating an oral diet and his IV fluids were stopped. His cultures remained negative. He remained afebrile.

On Day 4 he developed hyponatremia and his serum sodium levels declined to 126 mmol/L from 136 mmol/L. His urine sample was sent for urine osmolality, urine random chloride, urine random sodium, and blood sample for serum osmolality to identify the cause of hyponatremia. The results of his urine and serum osmolality are shown in Table 3. His TSH, blood glucose, serum triglyceride, serum cholesterol, and adrenocortical function testing including serum cortisol levels were within normal limits. Nephrology was consulted and these findings were consistent with the syndrome of inappropriate antidiuretic hormone secretion (SIADH). His SIADH was likely precipitated by his acute illness of MS. He was started on a fluid-restricted diet, furosemide tablet 20 mg oral daily and 1 g sodium chloride (salt) tablets twice daily. His serum sodium improved to 136 on Day 6.

**Table 3:** Urine studies and serum osmolality for hyponatremia workup.

Lab Parameter (units)	Result
Serum Sodium (mmol/L)	124
Serum Osmolality (mOsm/kg)	264
Urine Osmolality (mOsm/kg)	509
Urine Sodium Random (mmol/L)	163
Urine Chloride Random (mmol/L)	163

His MS and hyponatremia resolved and he was doing well. The patient was discharged in a stable condition.

## Discussion

MS in PD is usually caused by either an abrupt withdrawal or a reduction in the dosage of anti-parkinsonian drugs [5-7]. However, rare cases have been reported precipitation by infections, severe dehydration, or hot weather without any changes in anti-parkinsonian medications [2-5]. Our patient was taking his anti-parkinsonian drugs for PD as usual. His MS was likely precipitated by a recent change in his activities including working in hot weather and not drinking enough water. Ikebe et al. reported that MS occurs more frequently during summer [7].

The exact pathophysiology of MS in PD remains unknown; however, it is hypothesized that an impairment in the mesolimbic, hypothalamic, and nigrostriatal dopaminergic pathways might be involved in the pathogenesis of the syndrome [8]. 

The clinical features of MS include severe rigidity, hyperpyrexia, altered consciousness, dysautonomia, diaphoresis, tachycardia, and fluctuating blood pressure. Laboratory evaluation shows leukocytosis and elevated CK [9]. The clinical features of MS are essentially similar to those of NMS and it might be difficult to differentiate between the two. However, a thorough and detailed review of the history of the presentation of this condition can help differentiate the two diseases. NMS is usually precipitated by the use of antipsychotics or dopamine antagonists whereas MS is precipitated by abrupt withdrawal or reduction of dopaminergic drugs, infection, dehydration, or hot weather. Our patient likely did not have NMS because he was taking his medications as usual and had no changes to his medications. He had developed MS due to severe dehydration and working out in hot weather.

Treatment of MS consists of IV fluid hydration, external cooling to reduce high body temperature, and resumption of anti-parkinsonian drugs in the same amount as was taken by the patient before the onset of MS. The anti-parkinsonian drugs should be resumed as early as possible either orally or if needed by nasogastric tube [5-7]. If oral or nasogastric feeding is contraindicated due to problems like ileus, intravenous preparations should be used [10]. Our patient was given adequate IV fluid hydration, he did not require external cooling as he did not have any recurrent fever spikes and his home anti-parkinsonian drugs were restarted orally on Day 2 of admission, resulting in a quick recovery. Early detection and treatment of MS prevent mortality and prevents complications such as rhabdomyolysis, acute renal failure, aspiration pneumonia, and disseminated intravascular coagulation [5,6]. Two-thirds of patients recover if treatment is started early [6].

## Conclusions

MS is a potentially fatal complication that can occur during the course of drug treatment for PD and awareness of this condition is necessary for its prevention and successful treatment. Patients usually present with fever, altered levels of consciousness, rigidity, dysautonomia, leukocytosis, and elevated CK levels. The condition is easily confused with NMS due to similar clinical and laboratory manifestations of both diseases. However, careful and thorough history-taking can help differentiate MS from NMS. MS should be suspected whenever a patient with a history of PD presents with altered mentation, rigidity, fever spike, and dysautonomia. Treatment consists of ample IV fluid hydration, external cooling if needed, and resuming anti-parkinsonian drugs as early as possible to prevent mortality. 
